# Microbiome‐Immune Crosstalk in Inflammatory Skin Diseases: Mechanisms and Therapeutic Potential of Probiotics

**DOI:** 10.1002/iid3.70574

**Published:** 2026-10-29

**Authors:** Pranvi Sharma, Tanaka Nhokwara, Jaswinder Singh, Kiran Bala, Kajal Kamra

**Affiliations:** ^1^ Department of Microbiology, Gargi College Delhi University Delhi India; ^2^ Sally Mugabe Central Hospital Harare Zimbabwe; ^3^ Department of Emergency Medicine University of Nebraska Medical Center Omaha Nebraska USA; ^4^ Department of Zoology Deshbandhu College, University of Delhi Delhi India; ^5^ Department of Pharmaceutical Sciences University of Nebraska Medical Center Omaha Nebraska USA

**Keywords:** acne vulgaris, atopic dermatitis, gut‐skin axis, microbiome, probiotics, psoriasis, skin health

## Abstract

**Background:**

The gut–skin axis links intestinal microbial activity with inflammatory skin disease, yet findings across atopic dermatitis, acne vulgaris, and psoriasis remain inconsistent. This review examines how disease biology and host context shape microbiome‐related effects and responses to probiotics.

**Materials and Methods:**

Published clinical and experimental evidence on gut microbiota, immune and metabolic signaling, barrier dysfunction, inter‐organ communication, and probiotic interventions was narratively reviewed across atopic dermatitis, acne vulgaris, and psoriasis.

**Results:**

Microbiome‐related mechanisms differ by disease. In atopic dermatitis, they intersect with barrier dysfunction and altered immune regulation. In acne vulgaris, metabolic, hormonal, dietary, and neuroendocrine factors modify their effects. In psoriasis, gut dysbiosis occurs within a broader setting of systemic immune activation. Probiotic outcomes are similarly variable and depend on strain, host characteristics, disease context, and study design.

**Discussion:**

Gut–skin communication is better interpreted as part of an interconnected physiological network rather than a uniform linear pathway. Disease‐specific evaluation of microbiome changes and probiotic interventions may help explain conflicting findings and guide more targeted future studies.

## Graphical abstract

Schematic overview of the gut–skin axis highlighting how intestinal microbiota and gut‐derived signaling influence skin homeostasis through effects on barrier integrity, immune regulation, and enteroendocrine communication. Dysregulation of these pathways may contribute to skin barrier dysfunction, altered sebaceous and keratinocyte activity, and inflammatory skin disorders including atopic dermatitis, acne, and psoriasis.

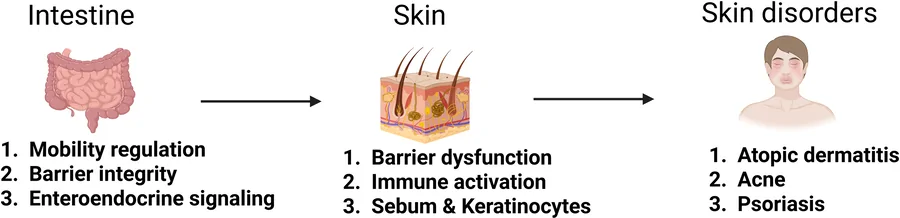

## 1 Introduction

The relationship between the gut microbiome and skin health has gained increasing attention over the past decade, with the term “gut‐skin axis” commonly used to describe this connection [1]. Clinical and experimental studies report associations between alterations in gut microbiota and atopic dermatitis, acne vulgaris, and psoriasis [2]. Earlier work helped establish that alterations in gut microbial composition may accompany inflammatory skin diseases, including atopic dermatitis, acne vulgaris, and psoriasis. These observations provided the foundation for testing microbiome‐based interventions, particularly probiotics, in dermatological settings (Table 1) [2, 3]. This broader perspective is particularly relevant when evaluating probiotics. Initially studied in gastrointestinal disease, probiotics are now tested in dermatology, but clinical outcomes vary by strain, disease biology, and study design. Some trials report improvement, while others show minimal or no benefit despite plausible mechanisms [4]. A major limitation in the field is the absence of a structured framework to interpret variability across studies. Findings are often presented in isolation, making it difficult to explain why similar microbial alterations produce different clinical outcomes across diseases or patient groups. This contributes to conflicting interpretations of probiotic efficacy [5, 6].

**Table 1:** Determinants of variability in microbiome and probiotic effects across dermatological diseases.

Factor	Atopic dermatitis	Acne vulgaris	Psoriasis
Dominant disease context	Barrier dysfunction with impaired immune tolerance	Metabolic and hormonal signaling (IGF‐1, mTOR), often diet‐related	Systemic immune activation (IL‐17/TNF‐driven inflammation)
How gut signals show up in disease	Changes in SCFA production and immune regulation can influence barrier integrity and inflammation	Gut‐related effects tend to act through metabolic pathways and inflammatory tone rather than directly on the skin	Associations with systemic inflammation; evidence for microbial translocation but variable clinical relevance
Where probiotics fit mechanistically	Can support barrier function and immune balance in some populations	Effects depend on diet, metabolic status, and baseline inflammation	Limited impact on core immune pathways driving disease
What clinical studies tend to show	Some improvement in severity scores, especially in pediatric cohorts	Mixed outcomes; small improvements in some studies, no effect in others	Modest or inconsistent changes in disease severity
Main reason results differ across studies	Differences in age, immune development, and baseline microbiome	Strong influence of diet, hormones, and lifestyle factors	Disease driven by complex immune networks that are not easily modified

However, current evidence remains difficult to interpret because similar microbial alterations do not produce uniform clinical outcomes across diseases or patient populations. Atopic dermatitis is closely linked to barrier dysfunction and altered immune tolerance, acne vulgaris is strongly influenced by metabolic and hormonal pathways, and psoriasis is dominated by systemic immune activation. Therefore, gut microbial changes may have different biological meanings depending on the disease context. This variability also helps explain why probiotic trials show inconsistent results, even when plausible mechanisms are proposed.

This review uses a comparative framework to examine gut microbiome associations and probiotic responses in atopic dermatitis, acne vulgaris, and psoriasis. Rather than treating the gut‐skin axis as a single linear pathway, we evaluate each disease according to observed microbiome findings, proposed biological pathways, strength of evidence, probiotic trial outcomes, and remaining uncertainty. This approach provides a clearer basis for understanding when microbiome‐targeted strategies may be clinically meaningful and when they are likely to remain adjunctive or context‐dependent.

## 2 Interpreting the Gut‐Skin Axis: Sources of Variability

In this review, microbiota refers to the living microbial communities present in the gut or skin, whereas microbiome refers to the collective genetic content and functional capacity of those communities. Many clinical studies use these terms interchangeably, but this distinction matters when sequencing, metagenomics, and personalized microbiome profiling are used to connect microbial taxa, microbial genes, and metabolic pathways with disease phenotypes [7]. To maintain consistency across the review, the disease‐specific sections are interpreted using the same sequence: observed microbiome findings, proposed biological pathway, strength of evidence, probiotic trial outcomes, and remaining uncertainty. Throughout this review, gut‐skin axis is used as the umbrella term for gut‐derived influences on skin disease, whereas gut‐brain‐skin, gut‐metabolic‐skin, and gut‐immune interactions refer to specific neuroendocrine, metabolic, and immune routes within this broader framework.

Research on the gut‐skin axis spans experimental and clinical settings, yet the findings are difficult to reconcile. Studies often report similar microbial shifts, but their associations with disease severity, progression, or treatment response vary widely [2, 8]. This points to a broader issue in how microbiome‐host interactions are interpreted, rather than a limitation of individual studies [3].

A major source of variability is the assumption that microbiome‐driven effects operate similarly across dermatological conditions [9]. Atopic dermatitis is characterized by barrier dysfunction and impaired immune tolerance, acne vulgaris is influenced by metabolic and hormonal signaling, and psoriasis involves systemic immune activation. The underlying biology of each disease determines how microbial alterations translate into clinical outcomes, meaning that similar dysbiosis patterns can have distinct consequences depending on the disease context [3, 9].

Host‐related factors further complicate interpretation. Age, diet, metabolic status, and environmental exposures influence baseline microbiome composition and immune responsiveness [10]. Pediatric and adult patients with atopic dermatitis often display different microbial signatures and treatment responses [11]. Dietary patterns associated with acne can alter metabolic signaling and microbial diversity. These variables are not consistently controlled or reported, which limits comparability across studies (Figure 1) [12, 13].

**Figure 1:**
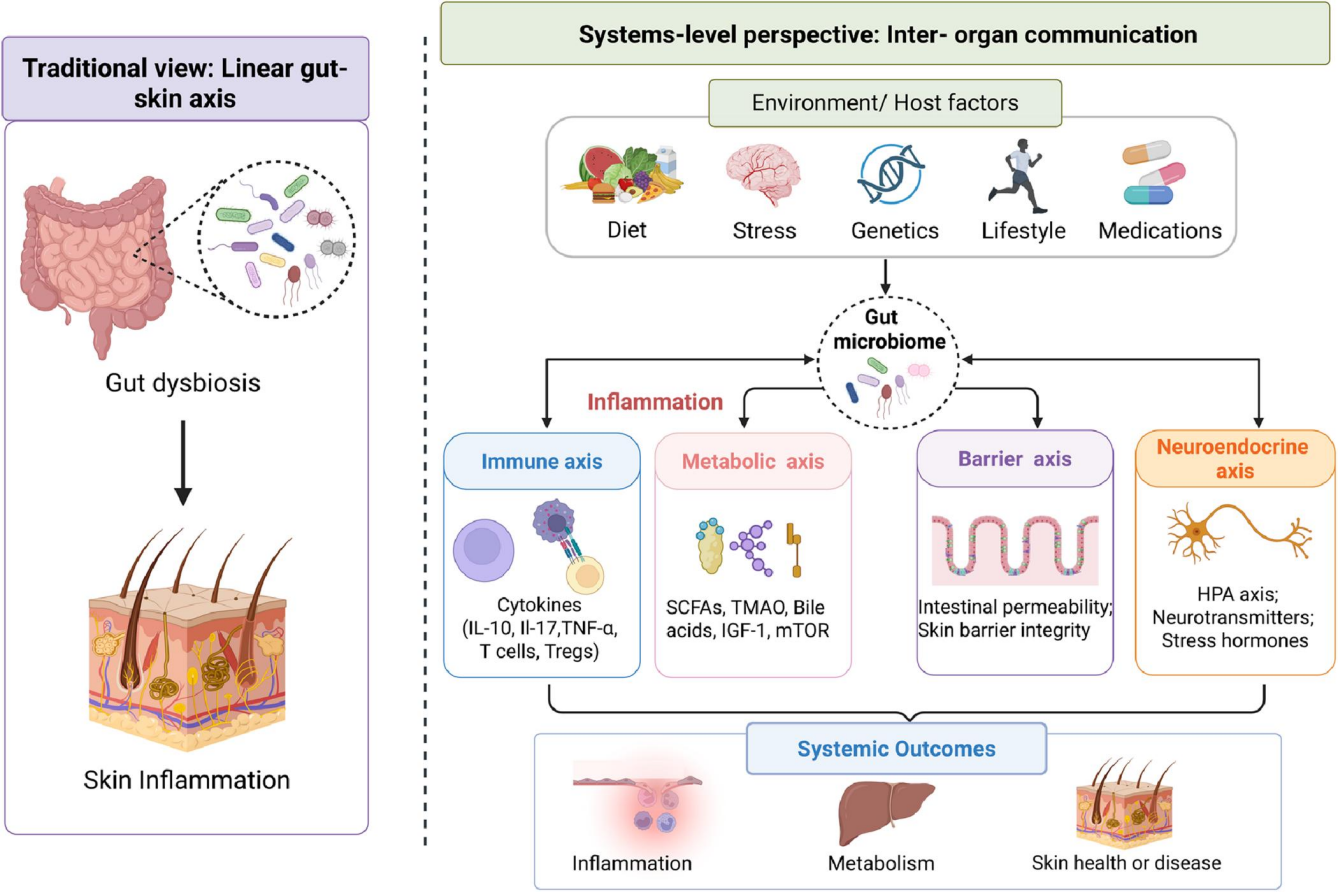
Systems‐level view of the gut–skin axis. The figure contrasts with the traditional linear model, in which gut dysbiosis directly promotes skin inflammation, with a broader systems‐level model. Environmental and host factors such as diet, stress, genetics, lifestyle, and medications influence the gut microbiome, which communicates with the skin through immune, metabolic, barrier, and neuroendocrine pathways. These interconnected axes collectively shape systemic inflammation, metabolism, and skin health or disease. Figure created using Biorender.com. HPA, hypothalamic–pituitary–adrenal; SCFAs, short‐chain fatty acids; TMAO, trimethylamine N‐oxide; IGF‐1, insulin‐like growth factor‐1; mTOR, mechanistic target of rapamycin.

Microbial heterogeneity adds another layer. Many studies describe changes at the genus or phylum level, yet functional differences between strains within the same species can be substantial [14]. This is especially relevant for probiotic interventions, where strains differ in their effects on barrier integrity, immune modulation, and metabolite production. Without strain‐level resolution, it becomes difficult to explain why some studies report benefit while others do not [3, 14, 15].

Study design also contributes to inconsistent findings (Table 2). Clinical trials vary in duration, sample size, and outcome measures, ranging from objective indices such as Scoring Atopic Dermatitis (SCORAD) and Psoriasis Area and Severity Index (PASI) to subjective symptom reporting [25, 26]. Short‐term studies may capture transient inflammatory changes without reflecting long‐term disease modification [27]. Small cohorts may not represent population‐level variability. As a result, interpreting findings across studies becomes difficult, particularly when comparing probiotic efficacy across studies.

**Table 2:** Representative clinical studies of probiotics in skin diseases.

Disease	Sample size and age/population	Exact strain/formulation	Design/duration	Key outcome	Reference
atopic dermatitis prevention	*n* = 159 mother–infant pairs randomized; *n* = 132 children evaluated at 2 years; high‐risk infants	*Lactobacillus rhamnosus* GG	RCT, high‐risk infants	Reduced early atopic eczema risk	[16]
atopic dermatitis treatment	*n* = 50 children; age 4–17 years; moderate atopic dermatitis	*B. lactis* CECT 8145, *B. longum* CECT 7347, *L. casei* CECT 9104	RCT	Reduced SCORAD and topical steroid use	[17]
atopic dermatitis treatment	*n*= 70 children/adolescents; age 4–17 years; 35 probiotic and 35 placebo	Probiotic mixture	Double‐blind placebo‐controlled RCT	Improved atopic dermatitis activity index/SCORAD	[18]
atopic dermatitis treatment	*n* = 100 children; age 6–36 months	*Lacticaseibacillus rhamnosus* GG	RCT, children with atopic dermatitis	Therapeutic effects in pediatric atopic dermatitis	[19]
Acne	*n* = 20 adults; 14 females and 6 males; mean age 33.7 ± 3.3 years	*Lactobacillus rhamnosus* SP1	Clinical supplementation study	Improved adult acne and insulin‐signaling gene expression	[20]
Acne	*n*= 74; age 12–30 years; 40 probiotic and 34 placebo	*Lacticaseibacillus rhamnosus* CECT 30031 + *Arthrospira platensis* BEA_IDA_0074B	12‐week randomized, double‐blind, placebo‐controlled trial	Improved clinical course of acne vulgaris	[21]
Psoriasis	*n* = 90 adults; age 18–70 years; plaque psoriasis	*B. longum* CECT 7347, *B. lactis* CECT 8145, *L. rhamnosus* CECT 8361	12‐week randomized, double‐blind, placebo‐controlled trial	Higher PASI75 response vs placebo	[22]
Psoriasis/inflammatory conditions	*n* = 26 psoriasis patients; age not stated in accessible abstract/table	*Bifidobacterium infantis* 35624	Placebo‐controlled human study	Reduced CRP and inflammatory cytokines, including in psoriasis cohort	[23]
Psoriasis	*n*= 27; 18 males and 9 females; age 22–67 years; mild‐to‐severe psoriasis vulgaris	*Bacteroides fragilis* BF839	Single‐arm open preliminary clinical study	Reduced PASI score and medication use	[24]

Abbreviations: CFU, colony‐forming units; CRP, C‐reactive protein; PASI, psoriasis area and severity index; PASI75, 75% reduction in psoriasis area and severity index; PGA, physician global assessment; RCT, randomized controlled trial; SCORAD, scoring atopic dermatitis.

Taken together, variability arises from interactions among disease biology, host environment, microbial composition, and study design. Interpreting microbiome‐related findings requires considering these factors together rather than in isolation (Figure 1). This integrated perspective provides the basis for examining disease‐specific patterns in the sections that follow.

## 3 Disease‐Specific Manifestations of Gut‐Skin Communication

To improve consistency across disease sections, each condition is interpreted using the same comparative sequence: observed gut microbiome findings, proposed biological pathway, strength of evidence, probiotic trial outcomes, and remaining uncertainty. This structure allows atopic dermatitis, acne vulgaris, and psoriasis to be compared without treating microbiome effects as uniform across diseases.

### 3.1 Atopic Dermatitis

Atopic dermatitis is often presented as a condition in which microbiome involvement is relatively direct, yet findings remain inconsistent across studies. The extent to which gut microbial alterations contribute to disease onset, severity, or treatment response varies, indicating that microbiome effects depend strongly on disease context and host characteristics (Figure 2).

**Figure 2:**
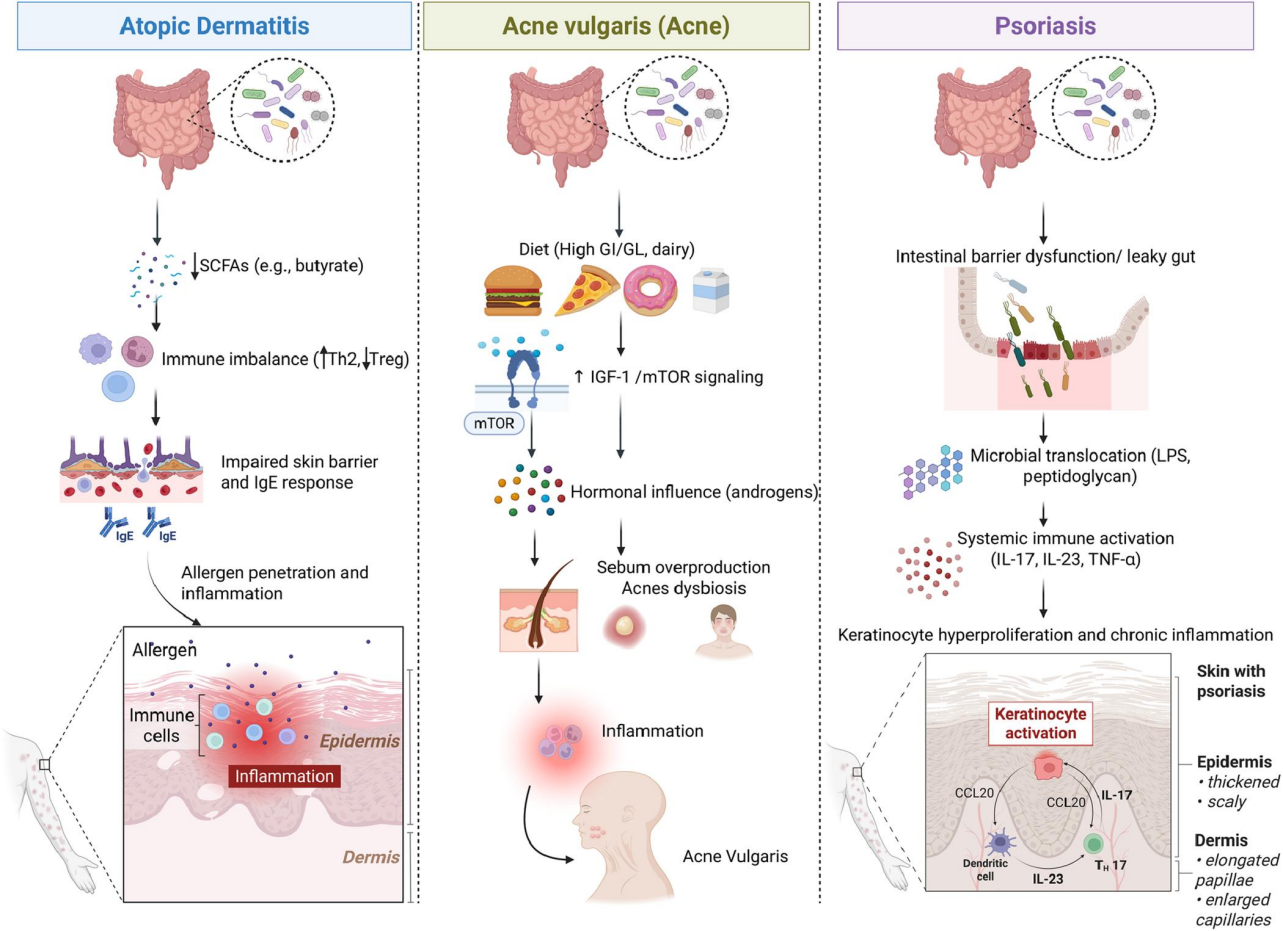
Disease‐specific patterns of gut–skin communication in inflammatory skin disorders. The figure summarizes distinct gut–skin axis mechanisms involved in atopic dermatitis, acne vulgaris, and psoriasis. In atopic dermatitis, reduced SCFAs contribute to immune imbalance, impaired skin barrier function, IgE responses, allergen penetration, and inflammation. In acne vulgaris, high glycemic‐load and dairy‐rich diets may enhance IGF‐1/mTOR signaling, hormonal activity, sebum production, and acne‐associated dysbiosis, promoting cutaneous inflammation. In psoriasis, intestinal barrier dysfunction enables microbial translocation, systemic immune activation, and IL‐17/IL‐23/TNF‐α–driven keratinocyte hyperproliferation and chronic inflammation. Figure created using Biorender.com. GI, glycemic index; GL, glycemic load; IGF‐1, insulin‐like growth factor‐1; IgE, immunoglobulin E; IL, interleukin; LPS, lipopolysaccharide; mTOR, mechanistic target of rapamycin; SCFAs, short‐chain fatty acids; TNF‐α, tumor necrosis factor‐alpha; Treg, regulatory T cell.

Atopic dermatitis has been associated with reduced levels of beneficial gut bacteria such as *Faecalibacterium prausnitzii* and *Bifidobacterium* species, along with increased levels of *Escherichia coli* and *Clostridioides difficile* [28, 29]. These shifts are commonly discussed in the context of immune imbalance, although their functional consequences differ across patient populations.

Barrier dysfunction is a defining feature of atopic dermatitis. While typically discussed in relation to the skin, intestinal barrier integrity may also contribute to disease activity [1]. Reduced production of short‐chain fatty acids and impaired immune tolerance have been proposed as mechanisms connecting gut dysbiosis to epithelial inflammation and heightened allergen sensitivity [30, 31]. However, not all patients exhibit the same microbial or metabolic patterns, which complicates attempts to define a single atopic dermatitis‐associated profile.

Immune regulation further contributes to variability. Atopic dermatitis involves altered immune tolerance and skewed T‐cell responses, but the degree to which gut‐derived signals influence these pathways differs across studies. Some reports show correlations between microbial alterations and disease severity, while others do not. Differences in host background, disease stage, and environmental exposures likely account for these inconsistencies, disease stage, and environmental exposures [32].

Clinical studies involving probiotics illustrate this variability. Some trials report improvements in severity indices such as SCORAD, while others show minimal or no benefit. Outcomes depend on factors such as strain selection, treatment duration, patient age, and baseline microbiome composition. Pediatric populations, where immune development is ongoing, may respond differently than adults. Even among similar study designs, results diverge, limiting generalization [33]. Overall, microbiome‐related effects in atopic dermatitis are not linear. Microbial composition, metabolite production, and immune signaling interact in ways that vary between individuals. Atopic dermatitis demonstrates that microbiome effects are shaped by disease biology and host context rather than a single uniform mechanism. Thus, atopic dermatitis shows the clearest alignment between gut microbial alteration, barrier dysfunction, immune modulation, and strain‐dependent probiotic response, although patient selection remains uncertain.

### 3.2 Acne Vulgaris

Acne vulgaris differs from atopic dermatitis in both its underlying drivers and the way microbiome‐related effects manifest (Figure 2). While barrier function contributes, acne is more strongly influenced by metabolic and hormonal signaling.

Acne is a multifactorial disorder in which insulin‐like growth factor‐1 (IGF‐1), forkhead box transcription factor O1 (FoxO1), and mammalian target of rapamycin (mTOR) play central roles. A study of 60 patients by Agamia et al. reported elevated serum IGF‐1 together with altered skin expression of FoxO1 and mTOR in individuals with acne compared with controls [34]. These findings place metabolic signaling at the center of disease activity and provide a framework for understanding how diet and systemic physiology influence severity [35].

The gut microbiome may influence these pathways indirectly [36, 37]. Dietary patterns associated with acne can alter microbial composition and metabolite production, which may affect insulin signaling and inflammatory tone [36, 38, 39]. Similar microbial profiles do not always correspond to similar clinical outcomes, suggesting that microbiome effects act as modulators rather than primary drivers [36, 37].

Dysbiosis and disruption may contribute to metabolic inflammation, although the magnitude of this contribution varies. Host factors such as hormonal status, diet, and baseline metabolic state influence whether these pathways become clinically relevant [38, 39].

The gut‐brain‐skin connection is also discussed in acne, particularly regarding stress and neuroendocrine signaling [8]. Psychological factors such as anxiety and depression can alter the gut microbiome and contribute to systemic inflammation, although the strength of this relationship differs between individuals [8]. Clinical studies involving probiotics reflect this variability (Table 2) [21, 40]. Some trials report reductions in lesion counts or improvements in severity, while others show minimal or no effect. Differences in study design, probiotic strains, and patient populations are likely to contribute [40]. Interventions aligned with metabolic and dietary context appear more consistent, suggesting that probiotic effects depend on the dominant disease pathways [21, 39, 40]. Compared to atopic dermatitis, where immune modulation and barrier support are more directly relevant, probiotic effects in acne are less predictable [40, 41, 42]. This reflects the broader range of contributing factors in acne pathogenesis [38, 39, 40]. Microbiome‐targeted strategies in acne, therefore, require a context‐dependent interpretation rather than a uniform expectation [36, 40].

Thus, acne vulgaris reflects a more indirect microbiome association, in which gut‐related effects appear to operate through metabolic, hormonal, and inflammatory pathways rather than through a primary barrier‐defect mechanism.

### 3.3 Psoriasis

Psoriasis represents a distinct pattern within gut‐skin interactions, with systemic immune activation playing a dominant role **(**Figure 2 **)** [43, 44]. Unlike atopic dermatitis and acne, psoriasis is driven primarily by immune signaling networks involving IL‐17 and TNF pathways [43, 45]. Psoriasis is a systemic autoimmune disease characterized by elevated levels of cytokines such as IL‐17, IL‐18, and IFN‐gamma [43, 45, 46, 47]. Alterations in gut microbiota composition, including reduced abundance of *Akkermansia muciniphila*, have been reported in patients with psoriasis [44, 48, 49, 50] [51]. These observations are consistent with a broader inflammatory state, although microbial signatures vary across studies [44, 49]. Intestinal permeability has received particular attention [44, 52, 53]. Bacterial DNA has been detected in the peripheral blood of patients with active psoriasis, suggesting microbial translocation and supporting a link between gut barrier disruption and systemic inflammation [54]. The extent of this contribution differs between individuals [44, 48, 52].

Despite these associations, translating microbiome findings into clinical outcomes remains challenging [55]. Microbial alterations are frequently observed, but their relationship to disease severity or progression is inconsistent [44, 48, 55]. Similar dysbiosis patterns also appear in other inflammatory conditions, limiting disease specificity [44, 55]. Probiotic interventions highlight this gap [56, 57, 58]. Experimental models show reductions in inflammatory markers and lesion severity following probiotic treatment, but human trials report mixed results [56, 57, 59, 60]. Some studies show modest improvements in PASI scores, while others show limited benefit [56, 57, 60]. Variability in study design, duration, and probiotic formulation contributes to these differences. Compared to atopic dermatitis and acne, psoriasis presents a scenario in which microbiome associations are relatively strong, but therapeutic modulation through probiotics is less consistent [44, 48, 56, 57, 58]. This supports a role for gut‐derived signals in disease activity but operates within a broader immune network that is not easily modified through microbiome‐targeted interventions alone. Thus, psoriasis shows strong microbiome‐immune associations, but probiotic responses remain less predictable because the dominant IL‐17/TNF‐driven inflammatory network may not be sufficiently modified by gut‐targeted intervention alone.

### 3.4 Cross‐Disease Comparison

The patterns observed across atopic dermatitis, acne vulgaris, and psoriasis show that microbiome involvement varies across dermatological conditions (Table 1) (Figure 2) [3, 61]. Similar categories of microbial alteration are reported, including dysbiosis, shifts in metabolite production, and immune activation, but the functional significance of these changes depends on the dominant biology of each disease [42]. Evidence across conditions supports the broader concept that gut and skin microbiota influence immune and metabolic pathways, although the direction and magnitude of these effects differ between diseases [62].

In atopic dermatitis, microbiome‐related mechanisms align more directly with core disease features such as barrier dysfunction and impaired immune tolerance [36, 42]. In acne vulgaris, microbiome effects intersect with metabolic and neuroendocrine pathways, where their contribution is more context‐dependent and less directly tied to primary disease drivers [36, 40]. In psoriasis, microbiome alterations are associated with systemic immune activation, but modifying the microbiome alone does not consistently alter disease trajectory [56, 58].

These differences shape how probiotic interventions perform across diseases. Conditions in which microbiome‐related pathways overlap with central disease mechanisms, such as atopic dermatitis, may show more consistent responses [42]. In contrast, diseases driven by broader systemic processes, such as acne vulgaris and psoriasis, tend to be less responsive to microbiome‐targeted strategies [56, 58].

## 4 Beyond the Gut–Skin Axis: Inter‐Organ Crosstalk in Context

Expanding the gut–skin axis to include other organ systems has become common in recent literature. The challenge is that these extensions are often presented as parallel pathways without clarifying when they meaningfully contribute to disease. In practice, inter‐organ crosstalk is not uniformly relevant. Its impact depends on whether it intersects with the dominant biological drivers of each condition.

### 4.1 Gut‐Brain‐Skin Interactions

The gut‐brain‐skin connection is most frequently discussed in relation to stress and neuroendocrine signaling (Figure 3) [8, 63]. Early observations linked emotional stress with acne severity, and subsequent work has shown that stress can alter gut microbial composition and systemic inflammatory markers. These changes may influence sebaceous gland activity and cytokine signaling, although the strength of this relationship varies across individuals [48, 64].

**Figure 3:**
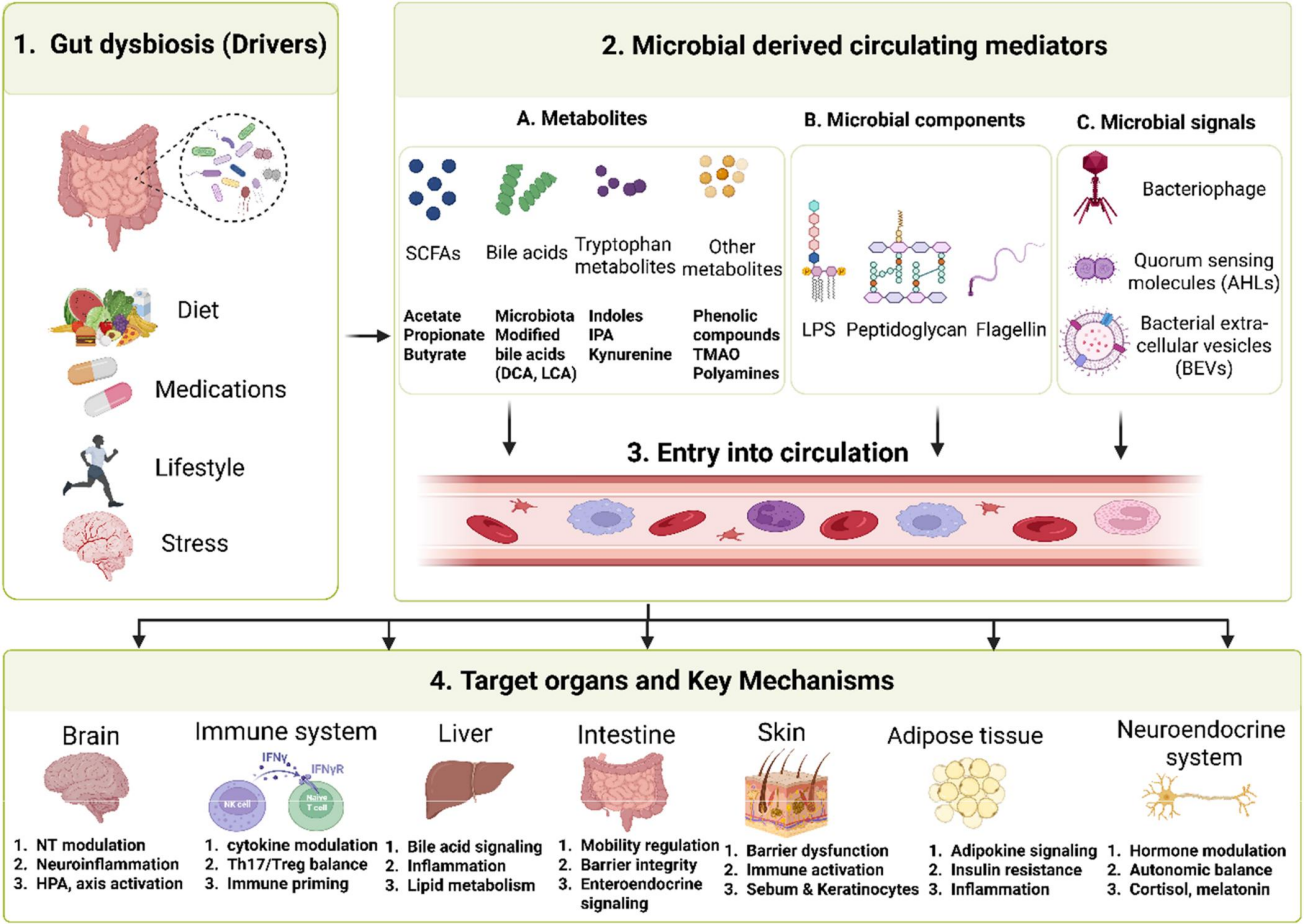
Circulating microbial mediators as drivers of inter‐organ crosstalk beyond the gut–skin axis. Gut dysbiosis, triggered by factors such as diet, medications, lifestyle, and stress, alters the production and release of microbial‐derived mediators. These include metabolites such as SCFAs, bile acids, tryptophan metabolites, TMAO, and polyamines; microbial components such as LPS, peptidoglycan, and flagellin; and microbial signals including bacteriophages, quorum‐sensing molecules, and bacterial extracellular vesicles. After entering the circulation, these mediators can act on distant organs, including the brain, immune system, liver, intestine, skin, adipose tissue, and neuroendocrine system. Through these pathways, gut dysbiosis may regulate immune balance, inflammation, metabolism, barrier integrity, neuroendocrine activity, and skin‐related immune responses. Figure created using Biorender.com. AHLs, acyl‐homoserine lactones; BEVs, bacterial extracellular vesicles; DCA, deoxycholic acid; HPA, hypothalamic–pituitary–adrenal; IPA, indole‐3‐propionic acid; LCA, lithocholic acid; LPS, lipopolysaccharide; NT, neurotransmitter; SCFAs, short‐chain fatty acids; TMAO, trimethylamine N‐oxide.

In acne vulgaris, this axis is supported by clinical observation and mechanistic plausibility [8, 64]. Stress‐related hormonal changes, including cortisol fluctuations, can influence sebum production and inflammatory tone [63]. Gut microbiome alterations associated with stress may further contribute to systemic immune activation [64]. Even so, not all individuals with stress‐related microbiome changes develop acne vulgaris flares, which highlights the role of host susceptibility and baseline metabolic context [8].

Mechanistically, stress activates the hypothalamo‐pituitary‐adrenal axis and sympathetic outputs, which can alter cortisol and corticotropin‐releasing hormone signaling. These signals may increase sebaceous lipogenesis, modify keratinocyte differentiation, and lower the threshold for inflammatory responses to Cutibacterium acnes. However, stress alone is not sufficient to cause acne in every individual. Clinical expression depends on baseline androgen and IGF‐1 activity, sebaceous gland responsiveness, diet, genetic susceptibility, age, existing C. acnes ecology, and the pre‐existing gut and skin microbiota.

In atopic dermatitis, stress is associated with increased pruritus and barrier disruption [63]. Neuroimmune signaling can amplify inflammatory responses, and gut‐derived signals may contribute through immune modulation [32, 65]. However, the degree to which gut microbiome changes drive these effects is not consistent across studies [8, 32].

For psoriasis, neuroendocrine influences are present but less central [8, 66]. Stress can exacerbate symptoms, but disease progression is more closely tied to sustained immune activation [66]. As a result, gut‐brain interactions are best understood as modifiers of symptom variability rather than primary drivers of disease course [8, 66].

### 4.2 Gut‐Metabolic‐Skin Interactions

Metabolic signaling represents a key interface between the gut microbiome and skin disease, particularly in acne vulgaris [36, 38, 39]. Dietary patterns associated with acne vulgaris, including high glycemic load and low fiber intake, influence systemic metabolism and microbial composition [36, 39]. These changes affect pathways such as IGF‐1 and mTOR, which are directly linked to sebaceous gland activity and keratinocyte proliferation (Table 1) (Figure 2) [34, 67]. Microbial metabolites also shape metabolic responses. Short‐chain fatty acids and other bioactive compounds can influence insulin sensitivity and inflammatory signaling [68]. In acne vulgaris, this creates a setting in which microbiome effects are present but mediated through metabolic pathways rather than acting directly on the skin [36, 39, 68]. This relationship helps explain why microbiome‐targeted interventions show inconsistent outcomes in acne vulgaris [68, 69]. When metabolic drivers remain unaddressed, changes in microbial composition alone may not produce measurable clinical effects [39, 69]. Studies that incorporate dietary modification alongside probiotic use tend to show more consistent improvements, suggesting that alignment with metabolic context is important.

In psoriasis, metabolic comorbidities such as obesity and insulin resistance are common [44]. Microbiome alterations may contribute to systemic inflammation in this setting, but their effects are intertwined with broader metabolic and immune processes [51]. This limits the impact of microbiome modulation as a standalone approach [44, 58, 68].

Atopic dermatitis is less strongly influenced by metabolic signaling. When present, metabolic factors tend to modify disease severity rather than act as primary drivers [70, 71].

### 4.3 Gut‐Immune System Integration

Immune signaling provides the most consistent link between the gut and the skin across dermatological conditions, although its role differs in scale and impact (Figures 2 and 3) [2, 3].

Under physiological conditions, gut microbial species and their products interact with immune cells through pattern‐recognition receptors, epithelial barrier signals, and metabolite‐sensitive pathways. Short‐chain fatty acids, indole derivatives, bile acid metabolites, and other postbiotic products can influence dendritic cell activity, regulatory T‐cell differentiation, Th17 responses, cytokine production, and epithelial barrier integrity [72]. Under dysbiosis or barrier dysfunction, these signals may shift from immune tolerance toward systemic inflammatory tone.

Figure 2 summarizes this context‐dependent immune model. It illustrates that microbiota‐related immune changes do not have the same meaning in every skin disease. In atopic dermatitis, microbial signals are closely aligned with barrier dysfunction and impaired immune tolerance. In acne vulgaris, immune activation is usually secondary to metabolic and hormonal pathways. In psoriasis, gut barrier dysfunction and microbial translocation may amplify systemic inflammation, but the dominant IL‐17 and TNF‐driven immune network remains difficult to modify through microbiome approaches alone.

In atopic dermatitis, immune dysregulation is closely tied to barrier dysfunction [41, 73]. Gut‐derived signals can influence T‐cell balance and cytokine production, which aligns with mechanisms targeted by certain probiotic strains [41]. This overlap may contribute to the measurable, though variable, clinical responses observed in some studies [41, 74].

In psoriasis, immune activation is systemic and sustained [2, 3, 44]. Evidence of increased intestinal permeability and the presence of microbial components in circulation supports a connection between gut barrier disruption and immune signaling (Figure 3) [46]. However, modifying the microbiome does not consistently alter the core immune pathways that drive disease, which limits therapeutic impact [2, 44].

In acne vulgaris, immune signaling is present but secondary to metabolic and hormonal influences. Microbiome‐related immune effects may contribute to inflammation, but they do not determine disease onset or progression [3, 36]. Inter‐organ crosstalk adds value only when it intersects with disease‐relevant pathways. Stress‐related signaling is more evident in acne vulgaris and atopic dermatitis, metabolic interactions are central in acne vulgaris, and immune integration is most pronounced in psoriasis [36, 62]. This context‐dependent relevance helps explain why microbiome‐targeted interventions produce different outcomes across diseases [74].

## 5 Do Probiotics Produce Disease‐Specific Clinical Benefit?

Probiotics are often presented as a straightforward extension of microbiome research, yet clinical results remain inconsistent. Improvements are reported in some settings, while others show minimal or no effect. Differences in disease biology, host context, and strain‐specific function help explain why probiotic outcomes vary across studies.

### 5.1 Mechanistic Targets of Probiotics

Probiotic effects are generally discussed in terms of three broad functions: modulation of barrier integrity, regulation of immune responses, and production of bioactive metabolites [75, 76]. These mechanisms are supported by experimental studies and reviews of probiotic–skin interactions [77]. Their clinical relevance depends on how closely they align with disease‐specific pathways [33, 40, 74].

In atopic dermatitis, these mechanisms overlap with central disease features [33, 74]. Barrier dysfunction and altered immune tolerance are key components of atopic dermatitis pathophysiology, and certain probiotic strains have been shown to influence epithelial integrity and cytokine balance. This alignment provides a plausible explanation for why some clinical studies report measurable improvement [33].

In acne vulgaris, probiotic effects are less directly aligned with dominant disease pathways. While probiotics may influence inflammatory tone and metabolic signaling, they do not directly target the hormonal and sebaceous drivers of disease [40]. This mismatch may contribute to the inconsistent outcomes observed across studies [40].

In psoriasis, probiotics may affect immune signaling and gut barrier function, but the disease is driven by a complex and sustained immune network. Modulating the microbiome alone is unlikely to produce large shifts in these pathways, which limits the magnitude of clinical response [58].

### 5.2 Strain‐Specific Effects and Functional Differences

One of the most common limitations in literature is the treatment of probiotics as a uniform category [15, 78]. In practice, effects are strain specific and cannot be generalized across species or genera [77]. Probiotic strains within genera such as *Lactobacillus* and *Bifidobacterium* have been studied for their ability to modulate immune responses, improve barrier integrity, and influence microbial balance [79, 80, 81]. Even within these groups, functional differences are substantial [15, 79]. Some strains produce higher levels of short‐chain fatty acids, while others are more active in regulating immune signaling pathways [80, 81].

Interpreting these results is challenging because of the variability of clinical studies [15, 40, 58]. Trials that use different strains, or combinations of strains, cannot be directly compared. In some cases, positive outcomes may be linked to specific functional properties that are not replicated in other formulations [15, 40, 82]. Without clear characterization of strain‐level effects, it becomes difficult to determine which probiotic interventions are likely to be effective [78, 82].

### 5.3 Clinical Outcomes and Sources of Variability

Clinical studies of probiotics in dermatological conditions show a wide range of outcomes [21, 40]. In atopic dermatitis, several trials report reductions in severity scores, particularly in pediatric populations, although heterogeneity across studies remains high [33, 83]. In acne vulgaris, results are mixed, with some studies showing modest improvements and others reporting no significant change [84]. In psoriasis, clinical data are limited, and reported effects are generally small or not statistically significant [56, 58]. This variability reflects differences in study design, patient characteristics, probiotic formulation, and external factors such as diet and medication use [56, 82].

The relationship between mechanistic plausibility and clinical outcome is not always direct [40, 56]. Interventions that influence immune signaling or metabolite production do not necessarily translate into measurable changes in disease severity. This gap highlights the importance of considering context when interpreting probiotic studies [82]. Therefore, probiotic effects should be interpreted in a disease‐specific rather than uniform manner [40, 56, 82]. Variability in clinical outcomes reflects these interacting factors rather than inconsistency in the underlying science [33, 56].

### 5.4 Atopic Dermatitis

Atopic dermatitis has the most developed clinical evidence base for probiotic interventions. Early interest in probiotic prevention strategies arose from studies suggesting that modulation of the infant gut microbiome might influence later eczema risk [33, 85]. However, randomized controlled trials evaluating probiotics for the treatment of established atopic dermatitis have produced mixed or negative results [83, 86, 87].

A placebo‐controlled trial of *Lactobacillus rhamnosus* GG in infants with mild to moderate atopic dermatitis found no therapeutic effect on SCORAD scores over a 12‐week period [87]. Symptom load improved over time in both groups, but there were no statistically significant differences in disease severity, rescue medication use, or immunologic markers [87].

Some studies using *Bifidobacterium* species or multi‐strain formulations have reported reductions in SCORAD scores, particularly in pediatric cohorts (Table 2) [17, 88]. At the same time, several trials using similar strains have failed to show significant benefit, underscoring the heterogeneity of clinical outcomes [33, 83, 87].

These differences are not easily explained by probiotic use alone [89]. Age appears to be an important factor, with more consistent effects reported in early‐life interventions than in older children or adults [85, 86, 89]. These findings suggest that probiotic benefit in atopic dermatitis is most likely when the intervention aligns with barrier dysfunction and immune regulation, although responses still differ between populations [33, 83, 89].

### 5.5 Acne Vulgaris

Clinical evidence for probiotics in acne vulgaris is more limited [40, 90]. A recent randomized clinical trial reported reductions in lesion counts and improvements in inflammatory markers over an 8‑week period [21]. Other smaller studies have shown modest improvements, particularly when probiotics are combined with standard therapies such as antibiotics or topical treatments [69, 84].

Not all trials show benefit. Some studies report minimal change in acne vulgaris severity despite similar durations of treatment [40]. These inconsistencies reflect the broader complexity of acne vulgaris pathogenesis [39]. Diet, hormonal status, and lifestyle factors influence outcomes and are not always controlled across studies [39].

Trials that incorporate metabolic context, such as dietary modification alongside probiotic use, tend to show more consistent results [39, 91]. This supports the idea that microbiome‑targeted interventions in acne vulgaris function within a larger metabolic framework rather than acting independently [39, 40, 90, 91].

### 5.6 Psoriasis

Clinical trials of probiotics in psoriasis are fewer and generally report modest effects [56, 58, 92]. In one randomized controlled study, patients receiving a multi‐strain probiotic formulation alongside topical therapy showed a greater reduction in PASI scores over 12 weeks (Table 2) [22]. Another study using a probiotic mixture reported small but measurable improvements in inflammatory markers [93]. These findings are not consistent across all studies [56, 58, 60, 92]. Some trials show no significant difference between probiotic and control groups [60]. Sample sizes are often small, and study durations vary, which limits the ability to draw firm conclusions [56, 58].

The pattern across trials reflects the underlying biology of psoriasis [44]. While microbiome alterations are associated with systemic inflammation, modifying the microbiome alone does not reliably alter disease course [44, 58, 92]. Probiotic interventions may have a role as adjunct therapies, but current evidence does not support their use as primary treatment.

## 6 Translational Implications, Future Directions, and Limitations

The variability observed across microbiome studies in dermatology reflects a broader issue in how these interventions are designed and interpreted [15, 82, 94]. Probiotics are often evaluated as uniform therapeutic agents, despite clear differences in strain function, disease biology, and host context. The mismatch between study design and biological context contributes to inconsistent clinical outcomes and limits translation into practice [82, 94].

One area that requires refinement is strain selection. Clinical studies illustrate this clearly. In atopic dermatitis, early work using *Lacticaseibacillus rhamnosus* GG (LGG) demonstrated reductions in eczema incidence in high‐risk infants (Table 2) [16]. More recent therapeutic trials, such as the ProPAD study, reported improvements in SCORAD scores and quality‐of‐life measures in children receiving LGG compared to placebo (Table 2) [19]. Later trials using different strains or combinations have shown mixed results. These differences are often attributed to study design, but they also reflect functional variation between strains [33, 83]. Future work needs to align strain selection with disease‐relevant mechanisms rather than treating probiotics as interchangeable.

Patient stratification is another major limitation. In pediatric atopic dermatitis, early‐life interventions tend to show more consistent effects than those in older populations, likely reflecting differences in immune development and microbiome composition [16, 33, 83]. In acne vulgaris, studies often include heterogeneous populations with varying dietary patterns and hormonal profiles, which can obscure treatment effects. A recent randomized clinical trial reported improvement in lesion counts following probiotic supplementation but variability across participants highlights the influence of baseline metabolic and environmental factors [21, 40].

Dietary and metabolic context also influences outcomes. In acne vulgaris, studies that incorporate broader metabolic considerations tend to show more consistent improvements than those evaluating probiotics in isolation [21, 39, 40]. These findings are consistent with the idea that microbiome‐targeted interventions function within a larger physiological environment rather than acting independently [40, 82, 94].

Study duration represents another limitation. Short‐term trials may capture changes in inflammatory markers or clinical indices without demonstrating sustained benefit [58, 83, 94]. A longer follow‐up is required to determine whether microbiome modulation leads to durable disease modification.

In psoriasis, the gap between mechanistic association and clinical response is evident. A randomized controlled trial using a multi‐strain probiotic formulation reported improvements in PASI scores, yet the magnitude of effect remains modest and not consistently replicated across studies. This reinforces the idea that microbiome modulation alone is unlikely to significantly alter complex immune‐driven disease processes [56, 58].

Beyond probiotics, there is growing interest in postbiotics and synbiotics as more targeted approaches [76]. These strategies may allow for more precise modulation of host–microbiome interactions, particularly when specific metabolites or pathways are involved. Their role in dermatological conditions remains underexplored [16, 76, 95].

Greater consistency in study design and reporting would also improve interpretability. Standardized outcome measures, clearer characterization of probiotic formulations, and better reporting of patient characteristics would facilitate comparison across studies and reduce ambiguity in conclusions.

## 7 Conclusion

The gut–skin axis has provided a useful starting point for understanding how the microbiome relates to dermatological disease, but its interpretation has often been simplified. Evidence across atopic dermatitis, acne vulgaris, and psoriasis shows that microbiome‐related effects are present, yet their clinical relevance differs across conditions.

These differences are not contradictory. They reflect variation in underlying disease biology, host factors, and the context in which microbial signals operate. In conditions such as atopic dermatitis, where barrier dysfunction and immune regulation are central, microbiome‐targeted interventions show more consistent effects. In acne vulgaris and psoriasis, where metabolic and systemic immune pathways play a larger role, responses are less predictable.

This perspective has implications for how microbiome research is interpreted. Probiotics should not be viewed as uniform therapeutic agents, and variability in clinical outcomes should not be taken as evidence against their relevance. Instead, these findings highlight the need to align microbiome‐targeted strategies with disease‐specific mechanisms and patient context.

A more selective and context‐driven approach may improve the translation of microbiome research into clinical practice. Rather than asking whether microbiomes influences skin disease in general, the more relevant question is when and under what conditions these effects become clinically meaningful.

## Author Contributions

**Pranvi Sharma:** writing – original draft, writing – review and editing, conceptualization. **Tanaka Nhokwara:** conceptualization, writing – original draft, writing – review and editing. **Jaswinder Singh:** conceptualization, writing – original draft, writing – review and editing, visualization. **Kiran Bala:** writing – review and editing, validation, conceptualization. **Kajal Kamra:** conceptualization, writing – original draft, writing – review and editing, project administration, supervision, visualization.

## Funding

The authors have nothing to report.

## Ethics Statement

Not applicable. This review article is based exclusively on previously published literature and did not involve any new studies with human participants or animals conducted by the authors.

## Consent

Not applicable. This review article is based exclusively on previously published literature and did not involve any new studies with human participants or animals conducted by the authors.

## Conflicts of Interest

The authors declare no conflicts of interest.

## Transparency Statement

Kajal Kamra affirms that this manuscript is an honest, accurate, and transparent account of the study being reported; that no important aspects of the study have been omitted; and that any discrepancies from the study as planned have been explained. All scientific content, references, interpretations, and final manuscript text were reviewed, verified, and approved by the authors. The authors take full responsibility for the integrity and accuracy of the manuscript.

## Data Availability

Data sharing not applicable‐ no new data generated, or the article describes entirely theoretical research.

## References

[1] L. Yang and J.‐N. Xia , “Beyond the Skin: Exploring the Gut‐Skin Axis and Metabolic Pathways in Atopic Dermatitis Pathogenesis,” International Journal of General Medicine 18 (2025): 6123–6136. 10.2147/IJGM.S550152 PMC12515402 41084738

[2] M. Jimenez‐Sanchez , L. S. Celiberto , H. Yang , H. P. Sham , and B. A. Vallance , “The Gut‐Skin Axis: A Bi‐Directional, Microbiota‐Driven Relationship With Therapeutic Potential,” Gut Microbes 17, no. 1 (2025): 2473524. 10.1080/19490976.2025.2473524 PMC11901370 40050613

[3] Y. Zhao , C. Yu , J. Zhang , Q. Yao , X. Zhu , and X. Zhou , “The Gut‐Skin Axis: Emerging Insights in Understanding and Treating Skin Diseases Through Gut Microbiome Modulation,” International Journal of Molecular Medicine 56, no. 6 (2025): 1–15. 10.3892/ijmm.2025.5651 PMC12494302 41041846

[4] V. Gowda , R. Sarkar , D. Verma , and A. Das , “Probiotics in Dermatology: An Evidence‐Based Approach,” Indian Dermatology Online Journal 15, no. 4 (2024): 571–583. 10.4103/idoj.idoj_614_23 PMC11265726 39050079

[5] R. J. Cano and G. García Menéndez , “Why Clinical Trials of Microbiome‐Targeted Interventions Often Fail to Support Health Claims: A Commentary on Probiotics and Translational Design,” Microorganisms 14, no. 2 (2026): 470. 10.3390/microorganisms14020470 PMC12942760 41753756

[6] R. L. Day , A. J. Harper , R. M. Woods , O. G. Davies , and L. M. Heaney , “Probiotics: Current Landscape and Future Horizons,” Future Science OA 5, no. 4 (2019): FSO391. 10.4155/fsoa-2019-0004 PMC6511921 31114711

[7] P. Nisa , A. V. Kirthi , and P. Sinha , “Microbiome‐Based Approaches to Personalized Nutrition: From Gut Health to Disease Prevention,” Folia Microbiologica 70, no. 5 (2025): 961–978. 10.1007/s12223-025-01337-6 40991166

[8] Z. Guo , J. Yang , R. Zang , Y. Yang , Q. Wang , and C. Xu , “The Brain–Gut–Skin Axis in Inflammatory and Disfiguring Skin Diseases: Mechanistic Insights, Clinical Correlations, and Therapeutic Strategies,” Frontiers in Immunology 17 (2026): 1737303. 10.3389/fimmu.2026.1737303 PMC12982022 41836406

[9] C. H. Lee , M. Min , S. S. Jin , and R. K. Sivamani , “Skin Microbiome Shifts in Various Dermatological Conditions,” Journal of Clinical Medicine 14, no. 17 (2025): 6137. 10.3390/jcm14176137 PMC12429258 40943897

[10] K. A. Klag and J. L. Round , “Microbiota‐Immune Interactions Regulate Metabolic Disease,” Journal of Immunology 207, no. 7 (2021): 1719–1724. 10.4049/jimmunol.2100419 PMC9105212 34544814

[11] B. Shi , N. J. Bangayan , E. Curd , et al., “The Skin Microbiome Is Different in Pediatric Versus Adult Atopic Dermatitis,” Journal of Allergy and Clinical Immunology 138, no. 4 (2016): 1233–1236. 10.1016/j.jaci.2016.04.053 PMC5235385 27474122

[12] R. C. Mareș , M. O. Săsăran , and C. O. Mărginean , “Gut Microbiota and Food Allergy: A Review of Mechanisms and Microbiota‐Targeted Interventions,” Nutrients 17, no. 18 (2025): 3009. 10.3390/nu17183009 PMC12472630 41010534

[13] A. Wilson , B. Quach , K. Musa , and I. Musa , “Gut Microbiome Dynamics in Food Allergy Development Across the Lifespan: Microbial Mechanisms, Host Interactions, and Therapeutic Perspectives,” Microorganisms 14, no. 5 (2026): 970. 10.3390/microorganisms14050970 PMC13209719 42197356

[14] Y. Zhao , Z. Ren , Q. Xu , et al., “Factors Influencing Gut Microbial Colonization: A Host‐Microbe‐Environment Interaction Perspective,” Current Research in Food Science 12 (2026): 101361. 10.1016/j.crfs.2026.101361 PMC12966602 41799257

[15] L. V. McFarland , C. T. Evans , and E. Goldstein , “Strain‐Specificity and Disease‐Specificity of Probiotic Efficacy: A Systematic Review and Meta‐Analysis,” Frontiers in Medicine 5, no. 124 (2018): 124. 10.3389/fmed.2018.00124 PMC5949321 29868585

[16] M. Kalliomäki , S. Salminen , H. Arvilommi , P. Kero , P. Koskinen , and E. Isolauri , “Probiotics in Primary Prevention of Atopic Disease: A Randomised Placebo‐Controlled Trial,” Lancet 357, no. 9262 (2001): 1076–1079. 10.1016/S0140-6736(00)04259-8 11297958

[17] V. Navarro‐López , A. Ramírez‐Boscá , D. Ramón‐Vidal , et al., “Effect of Oral Administration of a Mixture of Probiotic Strains on SCORAD Index and Use of Topical Steroids in Young Patients With Moderate Atopic Dermatitis: A Randomized Clinical Trial,” JAMA dermatology 154, no. 1 (2018): 37–43. 10.1001/jamadermatol.2017.3647 PMC5833582 29117309

[18] M. Feíto‐Rodríguez , A. Ramírez‐Boscà , S. Vidal‐Asensi , et al., “Randomized Double‐Blind Placebo‐Controlled Clinical Trial to Evaluate the Effect of a Mixture of Probiotic Strains on Symptom Severity and Use of Corticosteroids in Children and Adolescents With Atopic Dermatitis,” Clinical and Experimental Dermatology 48, no. 5 (2023): 495–503. 10.1093/ced/llad007 36637147

[19] L. Carucci , R. Nocerino , L. Paparo , et al., “Therapeutic Effects Elicited by the Probiotic Lacticaseibacillus Rhamnosus GG in Children With Atopic Dermatitis. The Results of the Propad Trial,” Pediatric Allergy and Immunology 33, no. 8 (2022): e13836. 10.1111/pai.13836 PMC9542056 36003050

[20] G. Fabbrocini , M. Bertona , Ó. Picazo , H. Pareja‐Galeano , G. Monfrecola , and E. Emanuele , “Supplementation With Lactobacillus Rhamnosus SP1 Normalises Skin Expression of Genes Implicated in Insulin Signalling and Improves Adult Acne,” Beneficial Microbes 7, no. 5 (2016): 625–630. 10.3920/BM2016.0089 27596801

[21] C. Eguren , A. Navarro‐Blasco , M. Corral‐Forteza , et al., “A Randomized Clinical Trial to Evaluate the Efficacy of an Oral Probiotic in Acne Vulgaris,” Acta Dermato‐Venereologica 104 (2024): adv33206. 10.2340/actadv.v104.33206 PMC11110809 38751177

[22] V. Navarro‐López , “Efficacy and Safety of Oral Administration of a Mixture of Probiotic Strains in Patients With Psoriasis: A Randomized Controlled Clinical Trial,” Acta Dermato‐Venereologica 99, no. 12 (2019): 1078–1084. 10.2340/00015555-3305 31453631

[23] D. Groeger , L. O'Mahony , E. F. Murphy , et al., “Bifidobacterium Infantis 35624 Modulates Host Inflammatory Processes Beyond the Gut,” Gut Microbes 4, no. 4 (2013): 325–339. 10.4161/gmic.25487 PMC3744517 23842110

[24] C. Lin , T. Zeng , Y. Deng , W. Yang , and J. Xiong , “[Treatment of Psoriasis Vulgaris Using Bacteroides Fragilis BF839: A Single‐Arm, Open Preliminary Clinical Study,” Sheng wu Gong Cheng xue bao= Chinese Journal of Biotechnology 37, no. 11 (2021): 3828–3835. 10.13345/j.cjb.210198 34841788

[25] Y. Liscano , D. Muñoz Morales , F. Suarez Daza , S. Vidal Cañas , D. Martinez Guevara , and E. Artunduaga Cañas , “Microbial Interventions for Inflammatory Skin Diseases: A Systematic Review and Meta‐Analysis of Atopic Dermatitis and Psoriasis,” Microorganisms 13, no. 11 (2025): 2416. 10.3390/microorganisms13112416 PMC12654813 41304102

[26] X. Xue , X. Yang , X. Shi , and Z. Deng , “Efficacy of Probiotics in Pediatric Atopic Dermatitis: A Systematic Review and Meta‐Analysis,” Clinical and Translational Allergy 13, no. 7 (2023): e12283. 10.1002/clt2.12283 PMC10349543 37488736

[27] W. Jiang , B. Ni , Z. Liu , et al., “The Role of Probiotics in the Prevention and Treatment of Atopic Dermatitis in Children: An Updated Systematic Review and Meta‑Analysis of Randomized Controlled Trials,” Pediatric Drugs 22, no. 5 (2020): 535–549. 10.1007/s40272-020-00410-6 32748341

[28] A. Acevedo‐Román , N. Pagán‐Zayas , L. I. Velázquez‐Rivera , A. C. Torres‐Ventura , and F. Godoy‐Vitorino , “Insights Into Gut Dysbiosis: Inflammatory Diseases, Obesity, and Restoration Approaches,” International Journal of Molecular Sciences 25, no. 17 (2024): 9715. 10.3390/ijms25179715 PMC11396321 39273662

[29] H. Song , Y. Yoo , J. Hwang , Y. C. Na , and H. S. Kim , “Faecalibacterium Prausnitzii Subspecies–Level Dysbiosis in the Human Gut Microbiome Underlying Atopic Dermatitis,” Journal of Allergy and Clinical Immunology 137, no. 3 (2016): 852–860. 10.1016/j.jaci.2015.08.021 26431583

[30] C. H. Kim , “Complex Regulatory Effects of Gut Microbial Short‐Chain Fatty Acids on Immune Tolerance and Autoimmunity,” Cellular & molecular immunology 20, no. 4 (2023): 341–350. 10.1038/s41423-023-00987-1 PMC10066346 36854801

[31] S.‐Y. Lee , E. Lee , Y. M. Park , and S. J. Hong , “Microbiome in the Gut‐Skin Axis in Atopic Dermatitis,” Allergy, Asthma & Immunology Research 10, no. 4 (2018): 354–362. 10.4168/aair.2018.10.4.354 PMC6021588 29949831

[32] B. Hou , H. Shao , D. Yuan , and E. H. Tham , “Skin and Gut Microbiome in Atopic Dermatitis: Mechanisms and Therapeutic Opportunities,” Pediatric Allergy and Immunology 36, no. 12 (2025): e70265. 10.1111/pai.70265 PMC12701371 41388767

[33] R. Gaikwad , S. Kondle , S. Chang , et al., “Impact of Probiotics on Atopic Dermatitis in Pediatric Patients: A Systematic Review and Meta‐Analysis,” Medicina 61, no. 12 (2025): 2090. 10.3390/medicina61122090 PMC12735213 41470092

[34] N. F. Agamia , D. M. Abdallah , O. Sorour , B. Mourad , and D. N. Younan , “Skin Expression of Mammalian Target of Rapamycin and Forkhead Box Transcription Factor O1, and Serum Insulin‐Like Growth factor‐1 in Patients With Acne Vulgaris and Their Relationship With Diet,” British Journal of Dermatology 174, no. 6 (2016): 1299–1307. 10.1111/bjd.14409 26799159

[35] P. Sánchez‐Pellicer , L. Navarro‐Moratalla , E. Núñez‐Delegido , B. Ruzafa‐Costas , J. Agüera‐Santos , and V. Navarro‐López , “Acne, Microbiome, and Probiotics: The Gut–Skin Axis,” Microorganisms 10, no. 7 (2022): 1303. 10.3390/microorganisms10071303 PMC9318165 35889022

[36] X. He , Z. Zhang , H. Jiang , et al., “Causal Association of Gut Microbes and Blood Metabolites With Acne Identified Through Systematic Mendelian Randomization,” Scientific Reports 14, no. 1 (2024): 26816. 10.1038/s41598-024-78603-z PMC11538280 39501024

[37] Y. Wu , X. Wang , W. Wu , and J. Yang , “Mendelian Randomization Analysis Reveals an Independent Causal Relationship Between Four Gut Microbes and Acne Vulgaris,” Frontiers in Microbiology 15 (2024): 1326339. 10.3389/fmicb.2024.1326339 PMC10869500 38371936

[38] O. E. Okoro , E. Camera , E. Flori , and M. Ottaviani , “Insulin and the Sebaceous Gland Function,” Frontiers in Physiology 14 (2023): 1252972. 10.3389/fphys.2023.1252972 PMC10505787 37727660

[39] I. Ryguła , W. Pikiewicz , and K. Kaminiów , “Impact of Diet and Nutrition in Patients With Acne Vulgaris,” Nutrients 16, no. 10 (2024): 1476. 10.3390/nu16101476 PMC11124289 38794714

[40] J.‐W. Tjiu and C.‐F. Lu , “Oral Probiotics in Acne vulgaris: A Systematic Review and Meta‐Analysis of Double‐Blind Randomized Clinical Trials,” Medicina 61, no. 12 (2025): 2152. 10.3390/medicina61122152 PMC12734792 41470154

[41] Z. Zhang , R. Wang , M. Li , and M. Lu , “Current Insights and Trends in Atopic Dermatitis and Microbiota Interactions: A Systematic Review and Bibliometric Analysis,” Frontiers in Microbiology 16 (2025): 1613315. 10.3389/fmicb.2025.1613315 PMC12237257 40630180

[42] X. Chen , “The Dual Role of Skin Microbiome Modulation in Precision Care for Atopic Dermatitis: A Review,” Health Sciences Review 17 (2025): 100245.

[43] A. W. Armstrong , A. Blauvelt , K. Callis Duffin , et al., “Psoriasis,” Nature Reviews Disease Primers 11, no. 1 (2025): 45. 10.1038/s41572-025-00630-5 40571687

[44] X. Zou , X. Zou , L. Gao , and H. Zhao , “Gut Microbiota and Psoriasis: Pathogenesis, Targeted Therapy, and Future Directions,” Frontiers in Cellular and Infection Microbiology 14 (2024): 1430586. 10.3389/fcimb.2024.1430586 PMC11335719 39170985

[45] L. Li , J. Liu , J. Lu , et al., “Interventions in Cytokine Signaling: Novel Horizons for Psoriasis Treatment,” Frontiers in Immunology 16 (2025): 1573905. 10.3389/fimmu.2025.1573905 PMC12037536 40303401

[46] L. Potestio , F. Martora , G. Lauletta , Y. Vallone , T. Battista , and M. Megna , “The Role of Interleukin 23/17 Axis in Psoriasis Management: A Comprehensive Review of Clinical Trials,” Clinical, Cosmetic and Investigational Dermatology 17 (2024): 829–842. 10.2147/CCID.S462797 PMC11016251 38616886

[47] A.‐M. Matei‐Man , C. Vesa , A. D. Pușcaș , et al., “Assessment of TNF‐α, IL‐12/23, and IL‐17 in Psoriasis: Only TNF‐α Reflects Clinical Response After 12 Weeks of Biologic Treatment,” Current Issues in Molecular Biology 47, no. 5 (2025): 368. 10.3390/cimb47050368 PMC12110024 40699767

[48] J. Chen , K. Sun , X. Zhang , et al., “Psoriasis and Gut Microbes: Research Advances From Mechanism to Therapy,” Frontiers in Microbiology 16 (2025): 1711288. 10.3389/fmicb.2025.1711288 PMC12712605 41425939

[49] K. Polak , T. Muszyński , A. Frątczak , et al., “Study of Gut Microbiome Alterations in Plaque Psoriasis Patients Compared to Healthy Individuals,” Advances in Dermatology and Allergology 41, no. 4 (2024): 378–387. 10.5114/ada.2024.142394 PMC11404103 39290901

[50] W. Huang , Y. Geng , J. Gong , and W. Wu , “The Impact of IL‐17A Inhibitors on Scalp and Gut Microbiota in Psoriasis,” Frontiers in Cellular and Infection Microbiology 15 (2025): 1623003. 10.3389/fcimb.2025.1623003 PMC12536032 41122690

[51] M. C. Buhaș , L. I. Gavrilaș , R. Candrea , et al., “Gut Microbiota in Psoriasis,” Nutrients 14, no. 14 (2022): 2970. 10.3390/nu14142970 PMC9321451 35889927

[52] M. Sikora , A. Stec , M. Chrabaszcz , et al., “Clinical Implications of Intestinal Barrier Damage in Psoriasis,” Journal of Inflammation Research 14 (2021): 237–243. 10.2147/JIR.S292544 PMC7851376 33542642

[53] A. Ramírez‐Boscá , V. Navarro‐López , A. Martínez‐Andrés , et al., “Identification of Bacterial DNA in the Peripheral Blood of Patients With Active Psoriasis,” JAMA Dermatology 151, no. 6 (2015): 670–671. 10.1001/jamadermatol.2014.5585 25760018

[54] F. M. Codoñer , A. Ramírez‐Bosca , E. Climent , et al., “Gut Microbial Composition in Patients With Psoriasis,” Scientific Reports 8, no. 1 (2018): 3812. 10.1038/s41598-018-22125-y PMC5830498 29491401

[55] M. Xue , Q. Deng , L. Deng , et al., “Alterations of Gut Microbiota for the Onset and Treatment of Psoriasis: A Systematic Review,” European Journal of Pharmacology 998 (2025): 177521. 10.1016/j.ejphar.2025.177521 40107339

[56] Y. Zhu , F. Xu , H. Chen , and Q. Zheng , “The Efficacy and Safety of Probiotics in the Adjuvant Treatment of Psoriasis: A Systematic Review and Meta‐Analysis of Randomized Controlled Trials,” Frontiers in Medicine 11 (2024): 1448626. 10.3389/fmed.2024.1448626 PMC11426359 39328313

[57] K. Wei , X. Liao , T. Yang , et al., “Efficacy of Probiotic Supplementation in the Treatment of Psoriasis—A Systematic Review and Meta‐Analysis,” Journal of cosmetic dermatology 23, no. 7 (2024): 2361–2367. 10.1111/jocd.16299 38551321

[58] L. Zhu , Y. Zhu , J. Xie , et al., “Efficacy and Safety of Gut Microbiota‐Targeted Therapy in Patients With Psoriasis: A Systematic Review and Meta‐Analysis of Randomized Controlled Trials,” BMC Immunology 26, no. 1 (2025): 72. 10.1186/s12865-025-00747-y PMC12465808 41013260

[59] X. Chen , Y. Chen , C. Stanton , et al., “Dose–Response Efficacy and Mechanisms of Orally Administered Bifidobacterium Breve CCFM683 on IMQ‐Induced Psoriasis in Mice,” Nutrients 15, no. 8 (2023): 1952. 10.3390/nu15081952 PMC10143451 37111171

[60] E. S. Suriano , M. Souza , C. M. Kobata , F. Santos , and M. J. Mimica , “Efficacy of An Adjuvant Lactobacillus Rhamnosus Formula in Improving Skin Lesions as Assessed by PASI in Patients With Plaque Psoriasis From a University‐Affiliated, Tertiary‐Referral Hospital in São Paulo (Brazil): A Parallel, Double‐Blind, Randomized Clinical Trial,” Archives of Dermatological Research 315, no. 6 (2023): 1621–1629. 10.1007/s00403-023-02553-1 36757438

[61] A. Ashkanani , G. Ashkanani , M. Yousef , et al., “Microbiome and Skin Health: A Systematic Review of Nutraceutical Interventions, Disease Severity, Inflammation, and Gut Microbiota,” Microorganisms 14, no. 1 (2025): 63. 10.3390/microorganisms14010063 PMC12844480 41597583

[62] B. De Pessemier , L. Grine , M. Debaere , A. Maes , B. Paetzold , and C. Callewaert , “Gut–Skin Axis: Current Knowledge of the Interrelationship Between Microbial Dysbiosis and Skin Conditions,” Microorganisms 9, no. 2 (2021): 353. 10.3390/microorganisms9020353 PMC7916842 33670115

[63] H. Zhang , M. Wang , X. Zhao , Y. Wang , X. Chen , and J. Su , “Role of Stress in Skin Diseases: A Neuroendocrine‐Immune Interaction View,” Brain, Behavior, and Immunity 116 (2024): 286–302. 10.1016/j.bbi.2023.12.005 38128623

[64] R. K. Sivamani , J. Maloh , and Y. Nong , “Correlating the Gut Microbiota and Circulating Hormones With Acne Lesion Counts and Skin Biophysical Features,” Microorganisms 11, no. 8 (2023): 2049. 10.3390/microorganisms11082049 PMC10459794 37630609

[65] Z. Zhang , C. Chang , L. Xiao , et al., “The Neuroimmune Axis in Atopic Dermatitis: From Pathogenic Mechanisms to Targeted Neuroimmunotherapy,” Journal of Inflammation Research 18 (2025): 18079–18113. 10.2147/JIR.S578036 PMC12746843 41473600

[66] H. Gao , Y. Fang , Y. Zhang , T. Xie , Z. Chen , and G. Wang , “Neuroimmune Crosstalk in Psoriasis: Mechanisms and Therapeutic Implications,” Inflammation 49 (2026): 14. 10.1007/s10753-025-02385-3 PMC12727763 41428266

[67] B. Melnik , “Linking Diet to Acne Metabolomics, Inflammation, and Comedogenesis: An Update,” Clinical, Cosmetic and Investigational Dermatology 8 (2015): 371–388. 10.2147/CCID.S69135 PMC4507494 26203267

[68] J. Xia , L. Ding , and G. Liu , “Metabolic Syndrome and Dermatological Diseases: Association and Treatment,” Nutrition & Metabolism 22, no. 1 (2025): 36. 10.1186/s12986-025-00924-1 PMC12057268 40329305

[69] P. V. Warp , S. M. Bilik , L. M. Ferrari , and J. E. Keri , “Prebiotics, Probiotics, and Postbiotics for Acne Vulgaris: A Systematic Review,” Dermatology and Therapy 16 (2026): 1531–1550. 10.1007/s13555-026-01659-4 PMC13013907 41703218

[70] M. Matwiejuk , H. Myśliwiec , A. Mikłosz , A. Chabowski , and I. Flisiak , “Is Atopic Dermatitis Associated With Systemic Metabolic Disturbances? A Systematic Review,” International Journal of Molecular Sciences 26, no. 12 (2025): 5884. 10.3390/ijms26125884 PMC12193471 40565347

[71] S. Traidl , M. M. Hollstein , N. Kroeger , et al., “Obesity Is Linked to Disease Severity in Moderate to Severe Atopic Dermatitis—Data From the Prospective Observational Treatgermany Registry,” Journal of the European Academy of Dermatology and Venereology 39, no. 1 (2025): 136–144. 10.1111/jdv.20042 PMC11664465 38661511

[72] K. A. Dogra , M. Sharma , N. Ghosh , G. Kaur , J. Singh , and P. Sinha , “Gut Microbiota and Immune Modulation: Role in Neurodegenerative Disorders and Cancer,” Molecular Biology Reports 53, no. 1 (2026): 935. 10.1007/s11033-026-12155-5 42307649

[73] M. Rios‐Carlos , D. Cervantes‐García , L. E. Córdova‐Dávalos , L. G. Bermúdez‐Humarán , and E. Salinas , “Unraveling the Gut‐Skin Axis in Atopic Dermatitis: Exploiting Insights for Therapeutic Strategies,” Gut Microbes 16, no. 1 (2024): 2430420. 10.1080/19490976.2024.2430420 PMC11610564 39601281

[74] A. E. Micu , I. A. Popescu , I. A. Halip , et al., “From Gut Dysbiosis to Skin Inflammation in Atopic Dermatitis: Probiotics and the Gut–Skin Axis—Clinical Outcomes and Microbiome Implications,” International Journal of Molecular Sciences 27, no. 1 (2025): 365. 10.3390/ijms27010365 PMC12785343 41516240

[75] J. Y. Hong , D. Kwon , and K. Y. Park , “Microbiome‐Based Interventions for Skin Aging and Barrier Function: A Comprehensive Review,” Annals of Dermatology 37, no. 5 (2025): 259. 10.5021/ad.25.009 PMC12505367 41044805

[76] S. K. Prajapati , L. Lekkala , D. Yadav , S. Jain , and H. Yadav , “Microbiome and Postbiotics in Skin Health,” Biomedicines 13, no. 4 (2025): 791. 10.3390/biomedicines13040791 PMC12025169 40299368

[77] T. Gao , X. Wang , Y. Li , and F. Ren , “The Role of Probiotics in Skin Health and Related Gut–Skin Axis: A Review,” Nutrients 15, no. 14 (2023): 3123. 10.3390/nu15143123 PMC10385652 37513540

[78] S. Binda , C. Hill , E. Johansen , et al., “Criteria to Qualify Microorganisms as “Probiotic” in Foods and Dietary Supplements,” Frontiers in Microbiology 11 (2020): 1662. 10.3389/fmicb.2020.01662 PMC7394020 32793153

[79] A. A. Churin , L. O. Sokolyanskaya , A. P. Lukina , and O. V. Karnachuk , “Current Concepts in Probiotic Safety and Efficacy,” Nutrients 18, no. 4 (2026): 696. 10.3390/nu18040696 PMC12942808 41754214

[80] P. Sankarganesh , A. Bhunia , A. Ganesh Kumar , A. S. Babu , S. T. Gopukumar , and E. Lokesh , “Short‐Chain Fatty Acids (SCFAS) in Gut Health: Implications for Drug Metabolism and Therapeutics,” Medicine in Microecology 25 (2025): 100139.

[81] Y.‐C. Yang , S. C. Chang , C. S. Hung , M. H. Shen , C. L. Lai , and C. J. Huang , “Gut‐Microbiota‐Derived Metabolites and Probiotic Strategies in Colorectal Cancer: Implications for Disease Modulation and Precision Therapy,” Nutrients 17, no. 15 (2025): 2501. 10.3390/nu17152501 PMC12348773 40806086

[82] L. V. McFarland , G. Hecht , M. E. Sanders , et al., “Recommendations to Improve Quality of Probiotic Systematic Reviews With Meta‐Analyses,” JAMA Network Open 6, no. 12 (2023): e2346872. 10.1001/jamanetworkopen.2023.46872 38064222

[83] M. I. Arif , Q. Dai , and L. Ru , “Probiotics for Pediatric Atopic Dermatitis: A Systematic Review and Meta‐Analysis of Randomized Controlled Trials,” Journal of Allergy and Clinical Immunology Global 5 (2026): 100649. 10.1016/j.jacig.2026.100649 PMC12887882 41675035

[84] L. Liang , X. Qi , X. Jiang , T. Chen , and L. Dong , “Lactobacillus Plantarum MH‐301 as An Effective Adjuvant to Isotretinoin in the Treatment of Acne Vulgaris: A Randomized and Open‐Label Trail,” Frontiers in Medicine 10 (2024): 1340068. 10.3389/fmed.2023.1340068 PMC10803606 38264057

[85] F. Wang , F. Wu , H. Chen , and B. Tang , “The Effect of Probiotics in the Prevention of Atopic Dermatitis in Children: A Systematic Review and Meta‐Analysis,” Translational Pediatrics 12, no. 4 (2023): 731–748. 10.21037/tp-23-200 PMC10167384 37181018

[86] M. A. Sjælland , M. T. Philipsen , T. B. Henriksen , J. Skipper , and S. Rubak , “Probiotics in Term Infants: Clinical Impact of Infant‐Type Bifidobacteria‐A Systematic Review and Meta‐Analyses,” Journal of Nutrition 155 (2025): 4075–4086. 10.1016/j.tjnut.2025.10.006 PMC12799449 41082980

[87] C. Grüber , M. Wendt , C. Sulser , et al., “Randomized, Placebo‐Controlled Trial of Lactobacillus Rhamnosus GG as Treatment of Atopic Dermatitis in Infancy,” Allergy 62, no. 11 (2007): 1270–1276. 10.1111/j.1398-9995.2007.01543.x 17919141

[88] P. D. S. M. A. Andrade , J. Maria e Silva , V. Carregaro , et al., “Efficacy of Probiotics in Children and Adolescents With Atopic Dermatitis: A Randomized, Double‐Blind, Placebo‐Controlled Study,” Frontiers in Nutrition 8 (2022): 833666. 10.3389/fnut.2021.833666 PMC8826069 35155534

[89] A. Xie , A. Chen , Y. Chen , et al., “Lactobacillus for the Treatment and Prevention of Atopic Dermatitis: Clinical and Experimental Evidence,” Frontiers in Cellular and Infection Microbiology 13 (2023): 1137275. 10.3389/fcimb.2023.1137275 PMC9978199 36875529

[90] M. Mohamed , A. Ullah , R. Hassan , M. Hamza , I. Mohamed , and M. Salam , “The Impact of Probiotics on Acne Vulgaris: A Meta‐Analysis of Randomized Controlled Trials,” Cureus 17, no. 11 (2025): e97010. 10.7759/cureus.97010 PMC12709052 41416302

[91] M. Luque‐Luna and M. Sidro‐Sarto , “RF‐The Role of Diet in the Management of Acne,” Actas Dermo‐Sifiliográficas 115, no. 7 (2024): T734–T736. 10.1016/j.ad.2023.06.026 38823770

[92] M. Waqas and I. Sarwar , “Efficacy of Probiotic Supplementation in the Management of Psoriasis: A Systematic Review,” Cureus 17, no. 12 (2025): e98265. 10.7759/cureus.98265 PMC12756675 41487796

[93] J. Moludi , H. Khedmatgozar , S. Saiedi , H. Razmi , M. Alizadeh , and B. Ebrahimi , “Probiotic Supplementation Improves Clinical Outcomes and Quality of Life Indicators in Patients With Plaque Psoriasis: A Randomized Double‐Blind Clinical Trial,” Clinical Nutrition ESPEN 46 (2021): 33–39. 10.1016/j.clnesp.2021.09.004 34857215

[94] R. A. Hillier , R. Gibson , T. Maruthappu , et al., “Probiotics, Prebiotics, and Synbiotics as Oral Supplements for Skin Health, Function, and Disease Throughout the Life Course: A Scoping Review,” Nutrition Reviews 84, no. 10 (2026): 2174–2192. 10.1093/nutrit/nuaf205 PMC13537454 41317036

[95] G. C. T. da Silva , K. de Pádua Silva , A. C. F. da Silva , et al., “Pre‐, Pro‐, Post‐, and Synbiotics for Skin Health: A Narrative Review of Cosmetic and Dermatological Applications,” Dermatological Reviews 7, no. 2 (2026): e70070.

